# Pulmonary metastasis of distal cholangiocarcinoma with multiple cavities in bilateral lungs: A case report

**DOI:** 10.1111/1759-7714.13584

**Published:** 2020-09-04

**Authors:** Nailiang Zhai, Jinping Liu, Pan Xu, Bo Liu, Yichu Fan, Changjun Lv

**Affiliations:** ^1^ Department of Pulmonary and Critical Care Medicine School of Medicine, Shandong University Jinan China; ^2^ Department of Pulmonary and Critical Care Medicine Binzhou Medical University Hospital Binzhou China; ^3^ Medicine and Pharmacy Research Center Binzhou Medical University Yantai China

**Keywords:** Cavity, computed tomography, distal cholangiocarcinoma, lymphatic metastasis, pulmonary metastatic

## Abstract

Cholangiocarcinoma is a type of malignant tumor derived from the epithelium of the bile duct. Cases of cholangiocarcinoma metastasis to the lung are rare, especially those with imaging features of multiple cavities in bilateral lungs. Here, we report a case of a patient who had previously undergone radical resection of primary distal cholangiocarcinoma 18 months ago. Transbronchoscopic lung biopsy of the right lung and biopsy of the left supraclavicular lymph node were performed for pathology confirmation, as well as immunohistochemistry. Multiple cavity shadows in bilateral lungs and enlarged lymph nodes were found on the computed tomography (CT) scan obtained 18 months postoperatively. No obviously enlarged lymph nodes were observed under the carina and beside the aortic arch, whereas enlarged lymph nodes were found above the left clavicle. Biopsy of lung and supraclavicular lymph nodes confirmed metastatic adenocarcinoma. Immunohistochemistry showed that it originated from the digestive tract and had the same homology as cholangiocarcinoma (CK19 +, Villin +). Cholangiocarcinoma can be transferred to the lung and the left supraclavicular lymph nodes through the lymphatic pathway by characteristic jumping lymph node metastases. Diffuse cystic change is a specific CT manifestation of the lymphatic lung metastasis of cholangiocarcinoma.

## Graphical abstract

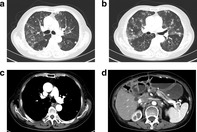

cystic cavity‐characteristic CT signs of translymphatic pulmonary metastasis in cholangiocarcinom.

## Introduction

Cholangiocarcinoma is a type of malignant tumor derived from the epithelium of the bile duct. Radical surgical resection is the only effective approach for treatment. However, cholangiocarcinoma can easily relapse after surgical resection, with a three‐year survival rate after surgery within 40%–60%,^1^ whereas the five‐year survival rate after surgery is lower than 20%.^2^ The lymph nodes or liver parenchyma around the bile duct are the most commonly metastasized sites of cholangiocarcinoma.^3^ It is noteworthy that distant metastases of cholangiocarcinoma are rarely visible.^4^ Pulmonary metastases are usually characterized by multiple, round, and various‐sized peripheral nodules (hematogenous metastasis) and diffuse small nodular thickening of the pulmonary interstitium (lymphatic metastasis).^5^, ^6^ The most common sites of primary tumors with supraclavicular lymph node metastasis are the lung, stomach, and esophagus, whereas the occurrence of cholangiocarcinoma is rare .^7^ Herein, we report a case of cholangiocarcinoma with bilateral lung and left supraclavicular lymph node metastases.

### Case report

**Figure 1:**
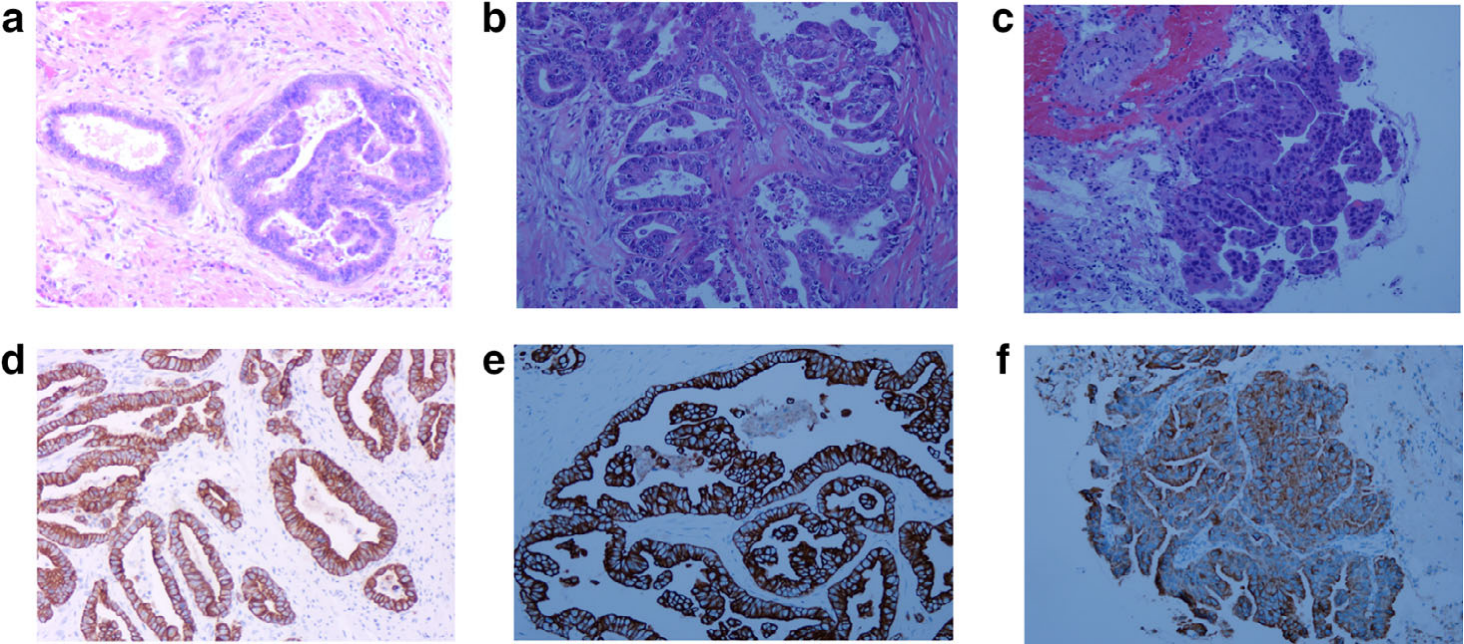
Hematoxylin and eosin (HE) staining and immunohistochemical staining of cancerous tissue in the bile duct, supraclavicular lymph node, and lung tissue. (**a**) HE staining of cholangiocarcinoma (10 × 10); (**b**) HE staining of the supraclavicular lymph node (20 × 10); (**c**) HE staining of the lung tissue (20 × 10); (**d**) cholangiocarcinoma, immunohistochemical staining showed positive CK‐19 and Tan under the microscope (10 × 10); (**e**) Immunohistochemical staining of lymph node showed positive CK‐19 and Tan under the microscope (20 × 10); (f) Immunohistochemical staining of the lung showed CK‐19‐ and Tan‐positive results under a microscope (20 × 10).

**Figure 2:**
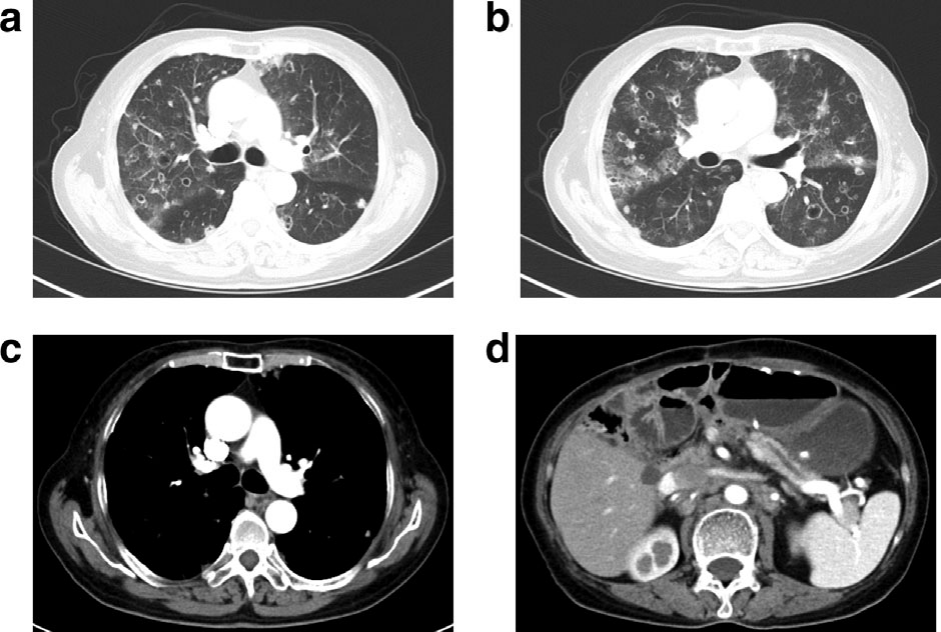
Enhanced CT image of chest and abdomen on 15 April 2018. (**a**, **b**) multiple cavitary shadows of bilateral lungs (arrow); (**c**) No enlarged lymph nodes were found under the carina or in the mediastinum; (**d)** Disordered structure of the pancreatic head area, dilated pancreatic duct, and gas accumulated in some intrahepatic bile ducts.

The patient was a 69‐year‐old woman of Han nationality. She was admitted to the Hospital with coughing that had lasted for two months which had been aggravated by fever for three days. She had a previous history of right breast cancer resection 16 years ago, with no history of smoking. She had a history of modified radical thyroidectomy for left thyroid cancer with partial thyroidectomy for right thyroid lobe for five years, and continuous oral administration of Euthyrox. Choledochectomy, pancreatectomy, and subtotal gastrectomy had previously been performed 15 months ago. Postoperative pathology examination showed moderately differentiated adenocarcinoma in the lower segment of the common bile duct and the ampulla of the duodenum (Fig 1a). Immunohistochemistry analysis showed CK19 (+) and Villin (+) (Fig 1d). No cancer was discovered at the cutting edge of the stomach, duodenum, common bile duct, and the pancreas. Metastasis was found in the lymph nodes around the pancreas (1/2), but not in the lymph nodes at the side of the greater curvature (0/8). A lymph node with a size of 1.0 × 1.0 cm was detected above the left clavicle, which was tough in texture, with good mobility, and without tenderness. Chest and abdomen computed tomography (CT) scans showed multiple striped, patchy, nodular, and ground‐glass high‐density shadows in bilateral lungs with fuzzy edges and burr in some of the nodules; obvious enhancement was observed in some of the nodules and there were multiple cavity shadows in bilateral lungs (Fig 2a,b), whereas no enlarged lymph nodes were visible in the mediastinum (Fig 2c). The structure of the pancreatic head was disordered. The dilated pancreatic duct revealed changes after partial gastrectomy, absence of the gallbladder, and multiple enlarged lymph nodes in the small curved side of the stomach and retroperitoneum (Fig 2d). Transbronchoscopic lung biopsy (TBLB) of the lateral basal segment of the inferior lobe of the right lung and biopsy of the left supraclavicular lymph node were then performed. Pathological evaluation showed (lymph node) metastatic adenocarcinoma, which was considered to have originated from the digestive tract. Immunohistochemistry showed CK7 (+), CK19 (+), Villin (+), TIF‐1 (−), and Napsin A (−) (Fig 1b,e); (right lung): metastatic adenocarcinoma, originated from the digestive tract, not excluding the bile duct (Fig 1c,f); immunohistochemistry: TIF‐1 (−), Napsin A (−), CK19 (+), Villin (+). The final diagnosis was cholangiocarcinoma with bilateral lung metastasis, and lymph node metastasis. Unfortunately, the patient did not continue with treatment and died one month after discharge.

## Discussion

The lower segment of cholangiocarcinoma is prone to vascular infiltration and early lymph node metastasis because it is surrounded by the portal vein, superior mesenteric artery and vein, and lymphatic vessels. The prognosis of this malignant tumor is closely related to the presence of lymph node metastasis^8^ and the positive rate of the metastatic lymph node (LNR).^9^ Multiple enlarged lymph nodes were found on the chest and abdomen CT scan 18 months postoperatively. Enlarged lymph nodes were also detected above the left clavicle, whereas no enlarged lymph nodes were visible under the carina and beside the aortic arch. Biopsy of supraclavicular lymph nodes confirmed metastatic adenocarcinoma. Immunohistochemistry showed that it originated from the digestive tract and had the same homology with cholangiocarcinoma (CK19 +, Villin +), thus suggesting that metastasis of peripheral lymph nodes and skip metastasis would occur at the early stage of lower cholangiocarcinoma.

Cavitary pulmonary metastasis often accompanies squamous cell carcinoma of the head, neck, and cervix, as well as gastrointestinal tract adenocarcinoma, gallbladder cancer, bladder cancer, and ovarian adenocarcinoma and osteosarcoma.^10^, ^11^, ^12^, ^13^, ^14^, ^15^ The chest CT findings in this patient were multiple annular cavities and a cystic cavity in bilateral lungs. TBLB confirmed metastatic lung adenocarcinoma, which was homogeneous with cholangiocarcinoma, thus suggesting that the lung metastasis of cholangiocarcinoma was pleomorphic. Therefore, a cystic cavity might be one of the characteristic CT signs of translymphatic pulmonary metastasis in cholangiocarcinoma.

## Disclosure

The authors of this work have nothing to disclose.

## Supporting information


**Figure S1**. Immunohistochemical staining of Villin in bile duct, supraclavicular lymph node and lung tissue. (a) Cholangiocarcinoma, immunohistochemical staining showed positive Villin and Tan under a microscope (10 × 10); (b) Immunohistochemical staining of supraclavicular lymph node showed Villin‐ and Tan‐positive results under a microscope (20 × 10); (c) Immunohistochemical staining of lung tissue showed Villin‐ and Tan‐positive results under a microscope (20 × 10).
