# Supplementary figures and images for: Pulmonary metastasis of distal cholangiocarcinoma with multiple cavities in bilateral lungs: A case report
Source: Thorac Cancer. 2020 Sep 4;11(10):2998–3000. doi: 10.1111/1759-7714.13584 (PMC7529578; doi:10.1111/1759-7714.13584)

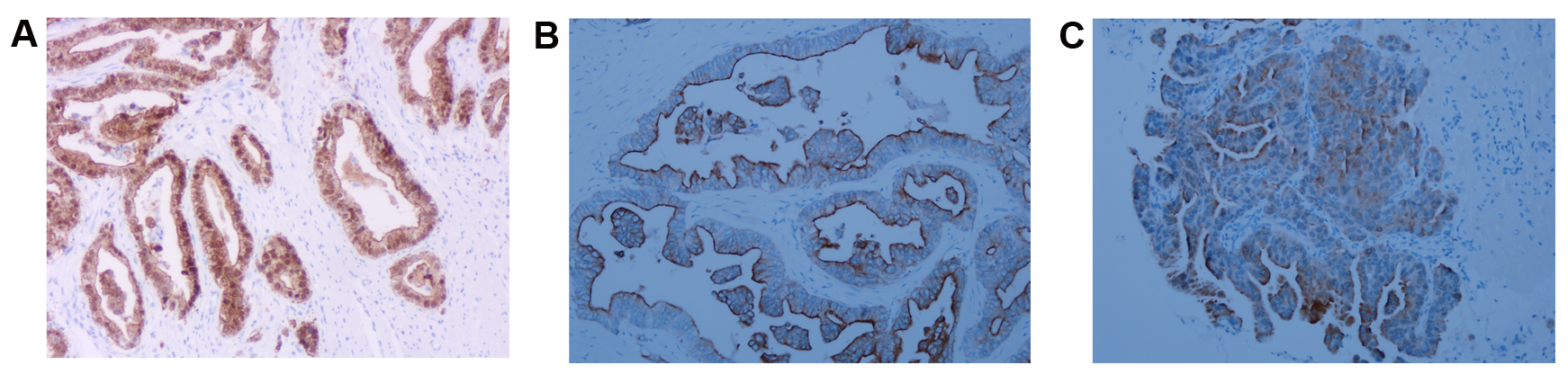

Supplement: Supplementary file 1 — Figure S1. Immunohistochemical staining of Villin in bile duct, supraclavicular lymph node and lung tissue. (a) Cholangiocarcinoma, immunohistochemical staining showed positive Villin and Tan under a microscope (10 × 10); (b) Immunohistochemical staining of supraclavicular lymph node showed Villin‐ and Tan‐positive results under a microscope (20 × 10); (c) Immunohistochemical staining of lung tissue showed Villin‐ and Tan‐positive results under a microscope (20 × 10). [file TCA-11-2998-s001.tif]
